# Associations of circulating irisin with 24-h blood pressure, total and visceral fat, and metabolic parameters in young adult hypertensives

**DOI:** 10.20945/2359-3997000000333

**Published:** 2021-02-25

**Authors:** Tomasz Miazgowski, Bartosz Miazgowski, Anna Kaczmarkiewicz, Jacek Kopeć

**Affiliations:** 1 Pomeranian Medical University Department of Propedeutics of Internal Diseases & Hypertension Szczecin Poland Department of Propedeutics of Internal Diseases & Hypertension, Pomeranian Medical University in Szczecin, Poland.; 2 Pomeranian Medical University Doctoral Study Szczecin Poland Doctoral Study, Pomeranian Medical University in Szczecin, Poland.; 3 University of British Columbia Biostatistics and Public Health Practice Division of Epidemiology Vancouver Canada Division of Epidemiology, Biostatistics and Public Health Practice, University of British Columbia, Vancouver, Canada.

**Keywords:** Irisin, visceral fat, 24-h blood pressure

## Abstract

**Objective:**

Some experimental and clinical studies suggest a possible role of irisin in central and peripheral regulation of blood pressure. The purpose of the study was to assess the associations between serum irisin levels, total and visceral fat, metabolic parameters, and blood pressure pattern during 24-h monitoring (ABPM).

**Materials and methods:**

In 206 patients with essential hypertension receiving standard antihypertensive treatments, we assessed anthropometric indices; serum irisin, blood lipids (total cholesterol, LDL-C, HDL-C, and triglycerides), glucose and insulin; body composition including lean mass and total, visceral, android and gynoid fat using a dual-energy x-ray absorptiometry; ABPM; and Homeostasis Model Assessment-Insulin Resistance (HOMA-IR).

**Results:**

Baseline irisin levels were within normal reference ranges and comparable between the genders. There were no significant correlations of irisin with age, anthropometric variables, lipids, HOMA-IR, body composition, as well as 24-h blood pressure and dipping status. In univariate analysis, age, fat mass and distribution, lipids and glucose, HOMA-IR, and nocturnal blood pressure fall were poor predictors of irisin levels. These neutral associations were not affected by age, gender, and treatment modality.

**Conclusions:**

In young adult hypertensives, serum concentration of irisin was within a normal range and not associated with total and regional fat, blood lipids, insulin resistance, as well as 24-h blood pressure and the magnitude of its nocturnal fall.

## INTRODUCTION

Irisin – a novel, exercise-induced, myokine produced by proteolytic cleavage of fibronectin type III domain-containing 5 (FNDC5) – is mainly involved in the regulation of energy metabolism by activating thermogenesis to increase energy expenditure (1). In addition, it has been suggested that the myokine might be associated with glucose and lipid metabolism, insulin sensitivity (2), oxidative stress (3), and variations in body weight (4). Some reports demonstrated an association of irisin with systolic (SBP) (5-7) and diastolic (DBP) (5-8) blood pressure, but other yielded conflicting results (9,10). In experimental rat models, administration of exogenous recombinant human irisin to the 3^rd^ brain ventricle activated neurons in the paraventricular nuclei of the hypothalamus resulting in the increases in SBP, DBP and cardiac contractibility. In contrast, when it was given intravenously, blood pressure was lowered but cardiac contractibility was not affected (11). These findings suggest that irisin can affect blood pressure by two different mechanisms: central, involving adrenergic sympathetic activity and increased vasopressin release, and peripheral – by a direct vasodilation through ATP-sensitive potassium channel and/or amelioration of endothelial dysfunction (11,12). Based on these observations, we theorized that that irisin might modulate diurnal blood pressure profile not only in rodents but also in humans. The purpose of this study was to investigate whether circulating irisin is independently associated with 24-h SBP, DBP, and physiological blood pressure nocturnal fall in young adult hypertensives. We also examined associations between irisin, metabolic parameters and fat distribution including visceral adipose tissue, which is known to contribute to many detrimental metabolic effects.

## MATERIALS AND METHODS

### Study participants

We included 206 young adult subjects (132 males; 74 females) aged 18-35 years who were diagnosed in our tertiary care unit with primary hypertension. The subjects with endocrine disorders, history of malignancy, nutrition disorders, and rapid weight changes (≥ 5 kg) within the last 12 months, as well as pregnant women and patients receiving medications or dietary supplements known to affect body composition (insulin, glucocorticoids, anabolic steroids, protein supplements, etc.) were excluded. Patients with secondary or spurious isolated systolic hypertension were also excluded. All subjects received standard antihypertensive drugs (13,14) including renin-angiotensin-aldosterone system (RAAS) inhibitors: angiotensin-converting enzyme or AT1 receptor inhibitors (men only; n=114); beta-blockers (n=42); calcium channel blockers (CCBs; n=100); thiazide-like diuretics (n=15); and alfa_1_-blockers (n=12) given either as a monotherapy or in combinations. The study complied with all applicable institutional regulations regarding the ethical use of human volunteers in research and the terms of the Declaration of Helsinki. The Pomeranian Medical University Ethics Committee approved the study protocol, and all participants gave their written consent.

### Procedures

Height (rounded to the nearest 0.5 cm), weight, waist circumference (WC) and hip circumference (HC) were measured. Body mass index (BMI) was calculated as BMI=weight/height^2^. Waist-to-hip ratio (WHR) was calculated as WHR=WC/HC. A 24-h ambulatory blood pressure monitoring (ABPM) was recorded using the Spacelabs device (model 90207; Spacelabs Healthcare; WA, USA). Automated blood pressure measurements were performed every 20 min during the day and every 30 min during nighttime (between 22.00 and 06.00). Based on a percent difference between daytime and nocturnal mean SBP, we identified the following blood pressure patterns: dipping (nocturnal SBP fall by 10-20%), non-dipping (<10%), extreme dipping (>20%) and reverse dipping (nocturnal SBP higher than daytime) (13,14).

### Biochemical assessment

After an overnight fast, lipid profiles including serum levels of triglycerides (TG) and total, low-density-lipoprotein (LDL-C)-, and high-density-lipoprotein (HDL-C)-cholesterol, glucose, and insulin were assessed. From insulin and glucose measurements, a Homeostatic-Model-Assessment Insulin Resistance Index (HOMA-IR) was calculated. Serum irisin was assessed by ELISA using recombinant antibodies (Irisin Recombinant Human, Mouse, Rat, Canine; Phoenix Pharmaceuticals Inc., USA; normal range provided by the manufacturer: 5.8 – 23.2 ng/mL).

### Body composition

Body composition was assessed using dual-energy X-ray absorptiometry (DXA) (GE Healthcare Lunar Prodigy Advance; Madison, WI, USA) using the automatic whole-body scan mode. We analyzed total body (TBF), android, and gynoid fat, as well as lean mass (LM), which in DXA is a surrogate measure of muscle mass. Visceral fat (VF) was computed by the CoreScan application dedicated to GE Healthcare DXA devices. Body composition parameters were analyzed using age-, gender-, race-, and instrument-specific reference values for VF (15,16) and TBF (17).

### Definitions

Traditional metabolic risk factors were defined using the following International Diabetes Federation diagnostic criteria for metabolic syndrome in populations of European descent (18): (a) WC ≥ 94 cm in men and ≥ 80 cm in women; (b) fasting glucose ≥ 100 mg/dL (≥ 5.6 mmol/L); (c) HDL-C < 40 mg/dL (< 1.3 mmol/L) in men and < 50 mg/dL (< 1.29 mmol/L) in women; and (d) TG ≥ 150 mg/dL (1.7 mmol/L). For the definition of high total cholesterol and LDL-C, we used the following cut-offs: > 200 mg/dL (> 5.17 mmol/L) and ≥ 115 mg/dL (2.97 mmol/L), respectively. From (19) we used the value of HOMA-IR of ≥2.5 as a marker for insulin resistance.

### Statistical analyses

Descriptive statistics included means ± standard deviation (SD) for continuous variables and frequency distributions for categorical variables. Variables with normal distribution were compared using parametric Student’s *t* tests; otherwise, non-parametric Mann-Whitney U-tests and Kruskall-Wallis tests were used. Differences in serum irisin levels between patients receiving monotherapy from the main three antihypertensive drug classes were compared using ANOVA. Correlations between pairs of quantitative variables were assessed using Pearson’s linear correlations or Spearman’s rho correlations for normally and non-normally distributed variables, respectively. Using a post-hoc analysis, the statistical power of the study with 206 subjects was sufficient to detect with 80% probability the true effect size of an association between quantitative variables corresponding to a correlation coefficient of 0.20. Statistical analyses were performed using Statistica (StatSoft, Poland; version 13.0).

## RESULTS

**Figure 1:**
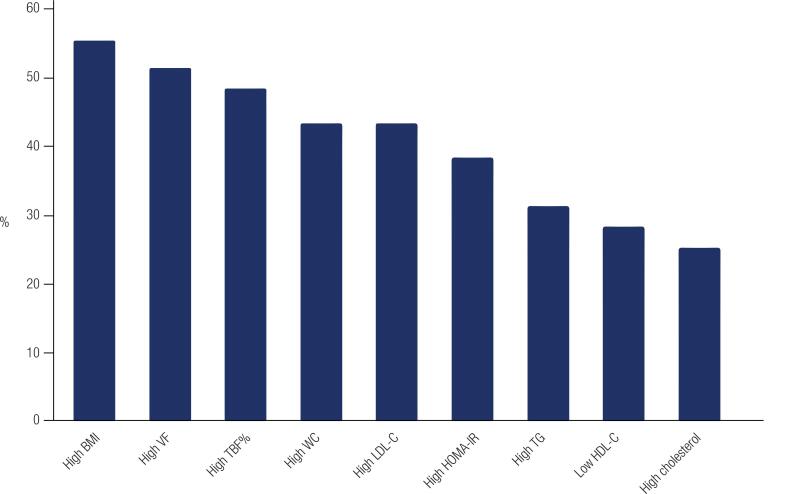
Frequency distribution of metabolic risk factors in the study population.

VF: visceral fat; TBF: total body fat; WC: waist circumference.

Patient characteristics are shown in Table 1 and Figure 1. Approximately 50% of patients had high BMI (> 25.0 kg/m^2^), WC, VF, and TBF. In approximately 20-25% of patients, mean values of SBP and DBP measured during a 24-h period, daytime and nighttime, were above recommended targets (13,14). In addition, almost 50% of males and 35% of females displayed a non-dipping pattern of blood pressure.

**Table 1:** Descriptive statistics of the study population

		All (n = 206)	Men (n = 132)	Women (n = 74)	P
Anthropometric variables					
Age (years)		27.40 ± 5.12	26.75 ± 5.13	27.55 ± 4.92	0.0509
Weight (kg)		83.24 ± 17.9	89.95 ± 14.6	71.27 ± 17.1	0.0001
Body mass index (kg/m^2^)		27.20 ± 4.90	27.84 ± 4.34	26.05 ± 5.73	0.0022
Waist circumference (cm)		93.99 ± 14.4	97.61 ± 13.1	87.54 ± 14.4	0.0001
Hip circumference (cm)		100.0 ± 11.1	96.62 ± 13.5	101.9 ± 9.03	0.0051
Waist-to-hip ratio		0.938 ± 0.10	0.955 ± 0.11	0.906 ± 0.10	0.0001
Family history of hypertension (n)		97 (47.08%)	60 (45.45%)	37 (50.0%)	0.6301
24-h Blood pressure					
Systolic blood pressure (mmHg)		131.2 ± 14.7	132.6 ± 14.7	128.7 ± 14.5	0.0519
	Daytime	135.8 ± 14.9	136.8 ± 14.9	133.9 ± 14.7	0.0601
	Nighttime	122.7 ± 13.7	123.4 ± 13.7	121.7 ± 13.6	0.0945
Diastolic blood pressure (mmHg)		77.31 ± 11.4	75.00 ± 10.0	81.43 ± 12.6	0.0014
	Daytime	83.24 ± 12.1	80.13 ± 11.9	85.90 ± 12.9	0.0142
	Nighttime	74.01 ± 11.3	72.06 ± 11.2	77.71 ± 11.4	0.0356
Nocturnal blood pressure fall (%)		-10.28 ± 4.81	-10.80 ± 4.73	-9.400 ± 4.92	0.0182
Biochemical assessment					
Insulin (µIU/mL)		12.59 ± 12.72	12.96 ± 15.01	11.89 ± 6.62	0.5121
Glucose (mg/dL)		88.96 ± 9.73	89.85 ± 9.90	87.32 ± 9.31	0.1180
HOMA-IR		2.853 ± 3.72	2.991 ± 4.52	2.598 ± 1.63	0.7751
Total cholesterol (mg/dL)		181.7 ± 36.7	186.5 ± 38.6	172.7 ± 31.1	0.0283
HDL-cholesterol (mg/dL)		51.86 ± 14.5	47.81 ± 12.8	59.38 ± 14.7	0.0001
LDL-cholesterol (mg/dL)		116.4 ± 33.9	122.2 ± 33.9	105.7 ± 31.2	0.0024
Triglycerides (mg/dL)		136.5 ± 71.3	147.9 ± 75.2	115.2 ± 58.1	0.0090
Irisin (ng/mL)		10.46 ± 3.23	10.25 ± 2.66	10.54 ± 2.68	0.3110
Body composition					
Total fat (kg)		26.54 ± 10.6	26.43 ± 10.0	26.73 ± 11.5	0.7750
Total fat (%)		32.42 ± 8.27	29.77 ± 7.38	37.14 ± 7.67	0.0001
Android fat (kg)		2.446 ± 1.41	2.571 ± 1.38	2.222 ± 1.43	0.0412
Gynoid fat (kg)		4.406 ± 2.26	4.101 ± 1.43	4.948 ± 2.27	0.0449
Visceral fat (kg)		0.920 ± 0.70	1.110 ± 0.71	0.580 ± 0.54	0.0001
Lean mass (kg)		53.73 ± 10.8	59.95 ± 7.23	42.63 ± 6.45	0.0001

P-value refers to comparison between women and men.

Serum irisin levels were within normal reference ranges and comparable between males and females. There were no significant differences in irisin levels between the groups receiving medications from three main antihypertensive drug classes in monotherapy (10.33 ± 2.62 ng/mL, 10.49 ± 2.71 ng/mL and 10.22 ± 2.76 ng/mL for RAAS inhibitors, CCBs, and beta-blockers, respectively). Similarly, differences in irisin serum concentrations between the patients with excess TBF% and VF defined as the values >1 SD from age- and gender-specific cut offs (for TBF%: >37.7% in females and >30.6% in males (17); for VF: >0.484 kg and >0.993 kg in females and males, respectively (15)), as well as between dippers and non-dippers were insignificant (Table 2).

**Table 2:** Serum irisin in relations to TBF, VF, BMI and dipping status

	Irisin (ng/mL)	P
TBF% within normal ranges (n = 108)	10.09 ± 2.54	0.3049
TBF% >37.7% (women); >30.6% (men) (n = 98)	10.46 ± 2.62	
VF within normal ranges (n = 103)	10.11 ± 2.49	0.2786
VF >0.484 kg (women); >0.993 kg (men) (n = 103)	10.50 ± 2.66	
Dippers (n = 123)	10.61 ± 2.64	0.2943
Non-dippers (n = 83)	10.23 ± 2.55	
BMI within normal ranges (n = 93)	9.831 ± 2.56	0.0737
BMI ≥25.0 kg/m^2^ (n = 113)	10.48 ± 2.60	

TBF: total body fat; VF: visceral fat; BMI: body mass index.

**Figure 2:**
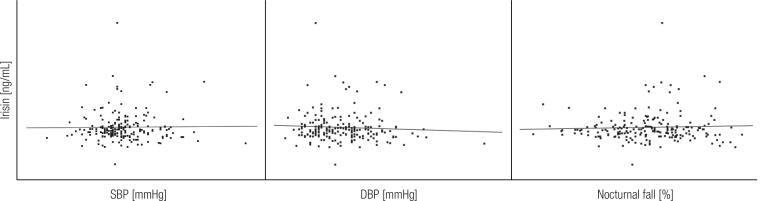
Correlations of irisin with systolic and diastolic blood pressure and nocturnal SBP fall.

**Figure 3:**
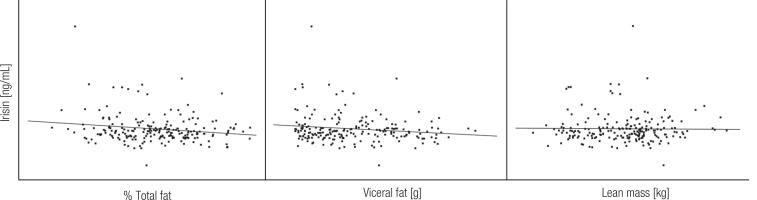
Correlations of irisin with total fat, visceral fat and lean mass.

In the whole group of patients (males and females combined), no significant correlations were found between irisin and ABPM (Figure 2) and body composition (Figure 3), as well as anthropometric measurements, blood lipids, insulin, glucose and HOMA-IR. Similarly, irisin was not associated with age, BMI, fat distribution, and other study variables in univariate regression (Table 3).

**Table 3:** Univariate regression analysis

Irisin (ng/mL)	β	SE	P
Age (years)	-0.0657	0.0626	0.2981
Body mass index (kg/m^2^)	-0.1899	0.4112	0.6456
Waist circumference (cm)	0.0510	0.1246	0.8319
Hip circumference (cm)	0.0800	0.2390	0.7398
24-h Systolic blood pressure (mmHg)	0.1256	0.3236	0.5621
24-h Diastolic blood pressure (mmHg)	0.1425	0.2986	0.4132
Nocturnal blood pressure fall (%)	0.1235	0.0322	0.2365
Insulin (μIU/mL)	0.1329	0.2566	0.0928
Glucose (mg/dL)	-0.1098	0.0999	0.1212
HOMA-IR	0.0009	0.7901	0.9900
HDL-cholesterol (mg/dL)	0.0388	0.0097	0.6935
LDL-cholesterol (mg/dL)	0.0325	0.2490	0.1967
Triglycerides (mg/dL)	0.0120	0.1280	0.3517
Total fat (%)	-0.1230	0.3180	0.6992
Android fat (kg)	0.0942	0.0689	0.1425
Gynoid fat (kg)	0.0067	0.0086	0.7224
Visceral fat (kg)	0.0203	0.0243	0.4074
Lean mass (kg)	0.0007	0.0005	0.8978

## DISCUSSION

The association of circulating irisin levels with blood pressure in humans has not yet been fully elucidated. Using different populations, some studies found irisin to be positively correlated with both SBP and DBP (5-7) or solely DBP (8), while other found no association (9,10). However, in all these reports, blood pressure was measured manually using automated devices, what in comparison of clinical studies can be a source of potential biases caused by random and systematic measurement errors (14,20-22). Instead, we measured blood pressure using ABPM method, which, in comparison to manual methods, is less influenced by inter-observer and inter-device variability. In addition, ABPM allows to perform a 24-hour measurement of blood pressure and its circadian patterns. Using ABPM, we found irisin not to be associated with SBP and DBP or the magnitude of SDP nocturnal fall. Moreover, these neutral associations were not affected by age, gender, and treatment modality. As this is the first study that assessed irisin associations with blood pressure measured by ABPM, we can only speculate that the myokine, which is released in response to intensive exercising, may exert only a very short-term rather than longer systemic effects on blood pressure, and hence – is not detectable in ABPM. This speculation might be supported by experimental data, in which acute central and peripheral administration of irisin that mimics exercise-induced irisin rise can temporary influence arterial blood pressure (11). In addition, intravenous injection of irisin in spontaneous hypertensive rats effectively reduced blood pressure likely *via* activation of nuclear factor E2-related factor-2 (23), suggesting its potential use in human hypertension.

We could not demonstrate the associations of irisin with body fat and its compartments, as well as obesity-dedicated measures such as BMI, waist and hip circumferences and waist-to-hip ratio. In addition, a novel finding of this study was that VF, which is known to be involved in the development of atherogenic lipid profiles, abnormal glucose tolerance, elevated blood pressure, and other abnormalities frequently related to metabolic syndrome (16,24) was not associated with irisin. Similarly, the myokine levels were not associated with glucose, insulin, HOMA-IR, and blood lipids. In earlier reports, both positive and negative correlations, as well as the lack of correlation between irisin concentrations and BMI, fat mass, lean mass, and other anthropometric parameters were found (6,25-27). Likewise, contradictory evidence was found for the association of irisin plasma levels with cardiovascular and metabolic parameters such as glucose, insulin, HOMA-IR, HDL-C, LDL-C, and TG levels in healthy individuals, as well as in those with obesity and the metabolic syndrome (4-6,9,10,26,27); however, it remains unclear whether these associations reflect a true cause-effect relationship.

Our study had some limitations. Firstly, the study is lacking a control group. This is because we investigated the association of irisin with nocturnal fall in blood pressure, and as non-dipping blood pressure is rare among healthy individuals, we focused on a group of young hypertensives, which comprised both dippers and non-dippers. Secondly, we did not assess the level of physical activity. However, although some studies proposed irisin to be an exercise hormone (28), other questioned the exercise-induced biological relevance of this myokine (25,29) suggesting that other, unknown factors may be involved in the regulation of exercise-induced irisin effects (30). Finally, we assessed irisin levels using a commercial ELISA kit but the accuracy of this method has been questioned. As human FNDC5, which is the precursor of irisin, is mainly translated from its non-canonical start codon, it has been claimed that human irisin antibodies commonly used in ELISA lack required specificity (31,32).

In summary, our study demonstrated that in young adults with hypertension serum concentration of irisin was normal and not associated with total and regional fat, blood lipids, insulin resistance, as well as 24-h blood pressure and its nocturnal fall.
